# Quality of Life and Philosophy of Life Determines Physical and Mental Health: Status Over Research Findings From The Quality of Life Research Center, Copenhagen, 1991-2007

**DOI:** 10.1100/tsw.2007.261

**Published:** 2007-10-22

**Authors:** Søren Ventegodt, Isack Kandel, Joav Merrick

**Affiliations:** ^1^ Quality of Life Research Center, Teglgårdstræde 4-8, DK-1452 Copenhagen K, Denmark; ^2^ Research Clinic for Holistic Medicine, Copenhagen, Denmark; ^3^ Nordic School of Holistic Medicine, Copenhagen, Denmark; ^4^ Scandinavian Foundation for Holistic Medicine, Sandvika, Norway; ^5^ Interuniversity College, Graz, Austria; ^6^ Faculty of Social Sciences, Department of Behavioral Sciences, Ariel University Center, Samaria, Ariel, Israel; ^7^ National Institute of Child Health and Human Development, Jerusalem, Israel; ^8^ Office of the Medical Director, Division for Mental Retardation, Ministry of Social Affairs, Jerusalem, Israel; ^9^ Kentucky Children's Hospital, University of Kentucky, Lexington, USA

**Keywords:** Quality of life, research, holistic health and medicine

## Abstract

Quality of life (QOL) has over the past decade become an important part of health science and also increased public awareness. It has become increasingly apparent that illness is closely related to the individual perception of a good life, and therefore the exploration of indicators related to quality of life appears to be of broad importance for the prevention and treatment of diseases. Identifying, which factors constitute a good life may reveal an understanding about what areas in life should be encouraged, in order to enhance the global quality of life, health, and ability. In this paper we present results from studies initiated in 1989 to examine quality of life in relation to disease. The purpose of this presentation was to assemble the results from the study carried out in the years between 1993 and 1997, examining a total of 11.500 Danes, to show the association between quality of life and a wide series of social indicators.

## INTRODUCTION

The concept of quality of life (QOL) has become an important topic both in the health field, social welfare and the political debate, especially in the Nordic countries. Enhancing the quality of life is more and more considered to be an objective in treatment and prevention of illness together with the provision of psychosocial support. For the last three decades QOL has been a major issue of debate in Denmark, and in recent years the concept of quality of life or living "a good life" has been the subject of a number of philosophical and psychological studies in Denmark made by other researchers before us (Aggernæs, 1989[1]; Henriksen, 1992[2]; Holm et al, 1994[3]; Kemp, 1991[4]; Sandøe, 1992[5]). It is becoming increasingly apparent that illness is closely related to the individual perception of a good life, and therefore the exploration of indicators related to quality of life appears to be of broad importance for the prevention and treatment of diseases.

## SEARCH FOR QUALITY OF LIFE

Our search for describing quality of life was built on the foundation that our quality of life must be composed of items that are known to us. Therefore a comparative valuation of these items must be our chief instrument for deciding the degree of quality of life of the person studied. The best quality of life will be the state that contains the greatest number of items having a positive value according to our own estimate. In order to arrive at correct decisions as to which items should be included in the questionnaire, it was necessary to consider what we would judge to be good and consider what comparative values to attach each item. From this approach we created first the SEQOL (self evaluation of quality of life) questionnaire, and later the much shorter and easier-used QOL1 and QOL5 questionnaires.

Identifying, which factors constitute a good life may reveal an understanding about what areas in life should be encouraged, in order to enhance the global quality of life, health, and ability. In this paper we present results from studies initiated in 1989 to examine quality of life in relation to disease. The purpose of this presentation is to assemble the results from the study carried out in the years between 1993 and 1997, examining a total of 11.500 Danes, to show the association between quality of life and a wide series of social indicators, in the ambition to make an almost complete map of QOL and the factors determining QOL, physical and mental health, and sexual, social and working ability (see the published series of papers below). We include three major lines of indicators: indicators of the present life, indicators of the personal history (life events), and indicators from the beginning of life, including a series of social and biological factors. Our belief was that a part of the variation of QOL in the adult population could be explained by biological and social heritage, another part could be explained by life event – happy and unhappy – and a third part could be explained by the conscious choices in life, recently, here and now.

Much to our surprise, data came out back in 1993-5 that convincingly showed us that our human consciousness seems to be the all-dominating factor that determines our quality of life, health, and ability. This discovery lead us to explore the possibilities for interviewing directly on the issue of human consciousness, and we found three major sources of knowledge on the intervention on the human consciousness for medical reasons: the classical holistic Hippocratic character medicine, which includes conversational therapy, bodywork, and spiritual exercises; the integrative and transcultural medicine that integrates all the medical systems of the pre-mordern cultures which most often also used conversational therapy, bodywork, and spiritual exercises with a major focus on bodywork and spiritual exercises, and the tradition of psychoanalysis and psychodynamic therapy, primarily conversational therapy but in some modern traditions also including bodywork.

In 1997 the Quality of Life Research Center took the initiative to establish an international research team on clinical holistic medicine (CHM) to meet the huge challenge of developing such a contemporary, medical synthesis aiming at integrating all existent consciousness-oriented (holistic) medical knowledge. Around the year 2000 the team had 20 members from three continents working together to create what today is called scientific or clinical, holistic medicine. Substantial funding from the IMK Almene Foundation though all the years – including the four-floor building called “The House of Health” that was created in central Copenhagen with funding of about 4 mill. EUROs, made this research possible. In the research centers at the House of Health we created one on theoretical psychosocial medicine and quality of life (The Quality of Life Research Center) and one on practical psychosocial, complementary and integrative medicine (The Researcxh Clinic for Holistic Medicine) and with this combination the new medical synthesis CHM was developed and tested on 500 volunteers most of them with severe, subjective, chronic health problems (physical, mental, existential and/or sexual issues), which could not be sufficiently helped by biomedicine (drugs or surgery).

## QUALITY OF LIFE RESEARCH CENTER

The Quality of Life Research Center, housing the Research Clinic for Holistic Medicine, is today a small, private research center, a NGO. It started at the University Hospital of Copenhagen (Rigshospitalet) and became busy in the early 1990s due to substantial funding from among other funds the The Pharmacist Foundation of 1991 and resources from the University hospital. In 1994 it became independent dedicated to understanding the connection between quality of life and health. Its mission is still research and development in quality of life, health and ability, and in the development of these crucial factors in life. To succeed with this hardly manageable mission, the center has from its beginning chosen to work with international networks, and in assuming an abstract and holistic approach to health sciences. The research up to 2004 is reviewed in an earlier paper [6].

## RESEARCH FROM THIS CENTER

The peer-reviewed papers and the scientific books from the QOLRCs research has been published in the following series:

1. QOL methodology (editorial, I-VII)[7-14] describing how global QOL can be measured.
2. QOL philosophy (editorial, I-VI)[15-21] suggesting a holistic way to re-think medical science.
3. QOL theory (I-III)[22-24] describing different ways to understand QOL scientifically.
4. QOL questionnaires (SEQOL, QOL5, QOL1)[25,26] is three validated tools to measure global QOL and subjective physical and mental health.
5. QOL results[27-37] is our statistical findings.
6. Theories of Existence (editorial, I-V)[38-46] is our speculations on the true nature of the human existence (or wholeness, or “soul”, or conscious roots of being)
7. Holistic medicine(I-IIII) [47-50]describes our research programs and methods for documenting effects of holistic healing, and the theory for holistic healing in individual therapy and a group setting.
8. QOL as medicine(I-III)[51-53] describes three pilot studies.
9. Clinical holistic medicine(I-VIXXX)[54-90] describes our new synthesis of Hippocratic, transcultural and psychodynamic approaches to consciousness-based medicine.
10. CHM: clinical results[91-96] is the first promising results from the clinical testing of scientific holistic medicine
11. Antonovsky and the sense of coherence[97-102] are 6 important papers testing the connection between sense of coherence and health.
12. Human Development(I-X)[103-112] is a new series of papers going into a deeper exploration of the nature of reality, trying to understand biological information and the connection between matter, life and consciousness.
13. Debate in the BMJ and Danish Medical Journal [113-127] discussed some of the clinical implications of our work. Only recent papers are listed.
14. Other papers[6,128,129] were papers on philosophy of science etc.
15. Scientific books on CHM[130-132] are from a new book series called “Principles of Holistic Medicine (Vol. I-III). Seven more volumes are planned.

In addition the Quality of Life Research Center has published about 10 popular books from the research, and in hundreds of long articles and thousands of small articles in the media/press it has been communicating its research to the public.

More than 100 peer reviewed scientific papers have now been published on quality of life and holistic medicine, making the Quality of Life Research Center one of the worlds most productive centres of new medical knowledge in the field of CAM and clinical holistic medicine – “quality of life as medicine”.

Since 2004 an international community has formed around the research and development of holistic medicine, the International Society for Holistic Health (ISHH) in 2004, and the Quality of Life Research Center has been the host of several international conferences. Through more than 1,000 presentations and lectures at most of the universities and large companies in Denmark the Quality of Life Research Center has made the public understand the fundamental importance of quality of life for health and prevention of illnesses.

After more than 1,000 presentations in the national medias, from the nine a clock news to national TV- and radio programs and the front pages of the leading news papers during the past 14 years, the Danish society at large now seems to have assimilated the key message: that you can improve your QOL and thus prevent disease and even improve your health and ability if you are sick, unhappy or poorly functioning.

## THOUGHTS FOR FUTURE PROJECTS

The most interesting future research project is the the Health Hospital, which is now open for funding; it will take the scientific holistic medicine to the next level, which is the phase 3 clinical studies, documenting the effect of the new drug-free clinical holistic medicine on a large number of patients with many different diseases from cancer, autoimmune diseases, HIV, and chronic pains to schizophrenia and existential, developmental and psycho-sexual problems. 40% of the Danish population – and the number is pretty much the same in the other European countries and the USA - is suffering from a chronic disease not being cured by biomedicine; we believe as holistic medicine seem to be able to help many of these patients[91-96] that around 2030 most patients will use holistic training programs to prevent and cure diseases instead of just taking bio-medical drugs, which most often is not effective with chronic illness. This is in accordance with the statistics documenting the exponential development in the Danish population’s use of CAM and holistic medicine: 400.000 Danes used it in 1990, and 800.000 Danes used it in 2000[133]. If this development continues CAM will be more popular that biomedicine around 2020. It is important to conduct the research now that will make us able to provide efficient and safe holistic medical treatments in the future to the many severely, chronically ill patients that are turning towards scientific holistic medicine and scientific CAM as their last hope.

We therefore sincerely hope that research in holistic medicine and the development of quality of life and health through interventions on the patients’ consciousness will be given high priority by governments, private funds and commercial sponsors. We need as the first important step forward with a Holistic Research Hospital, which can be established for around 100 mill. EUROs.

## ACKNOWLEDGEMENTS

The Danish Quality of Life Survey and the Quality of Life Research Center was 1991-2004 supported by grants from the 1991 Pharmacy Foundation, the Goodwill-fonden, the JL-Foundation, E. Danielsen and Wife's Foundation, Emmerick Meyer's Trust, the Frimodt-Heineken Foundation, the Hede Nielsen Family Foundation, Petrus Andersens Fond, Wholesaler C.P. Frederiksens Study Trust, Else & Mogens Wedell-Wedellsborg's Foundation and IMK Almene Fond. The research was approved by the Copenhagen Scientific Ethical Committee under number (KF)V.100.2123/91 and later correspondance.
